# Supplementary figures and images for: Spatially distinct FRL and Ena dependent actin networks coordinate nuclear positioning in Drosophila nurse cells
Source: PLoS Genet. 2026 Feb 9;22(2):e1012042. doi: 10.1371/journal.pgen.1012042 (PMC12915960; doi:10.1371/journal.pgen.1012042)

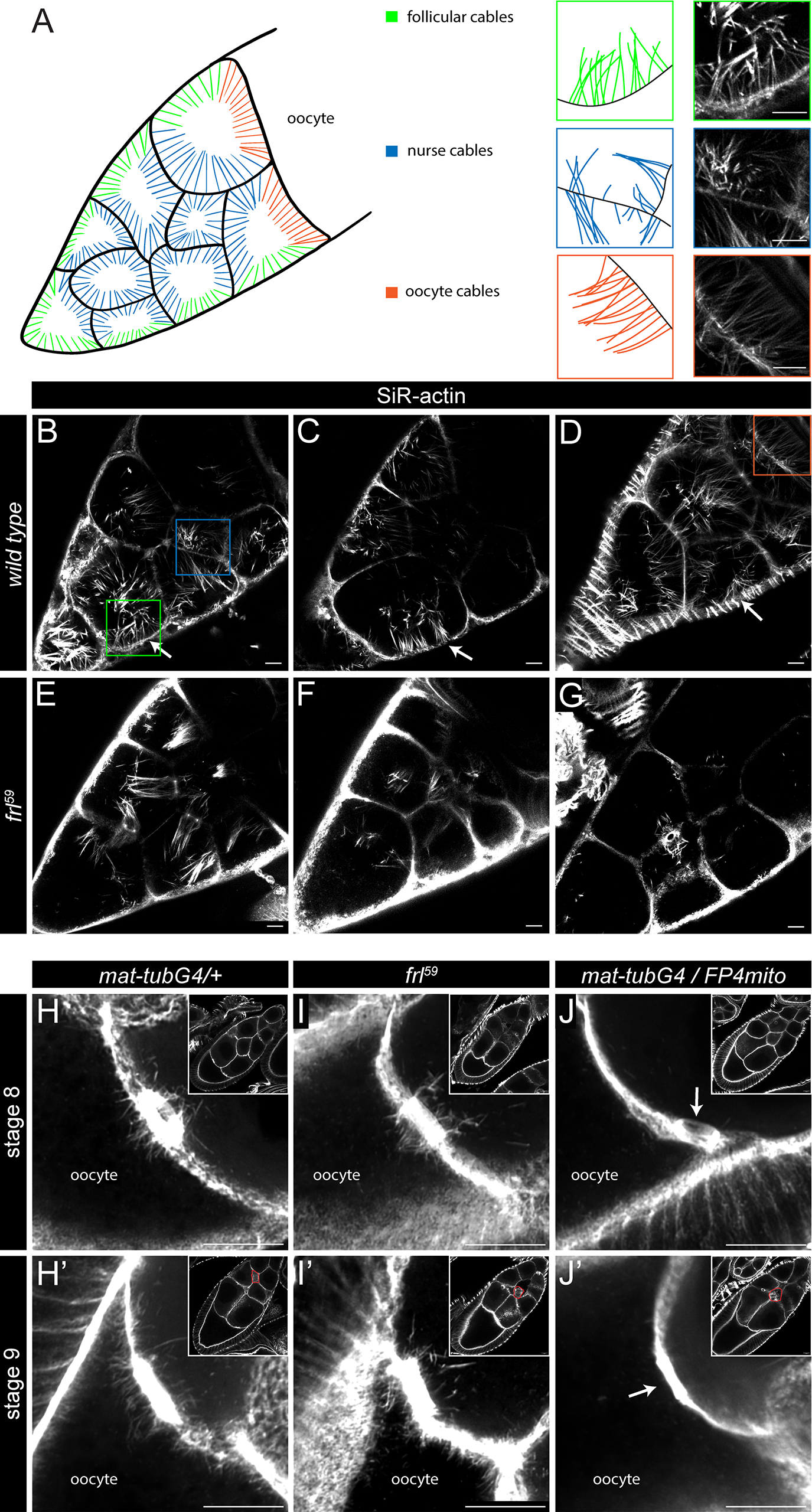

Supplement: S1 Fig — (TIF) [file pgen.1012042.s001.tif]

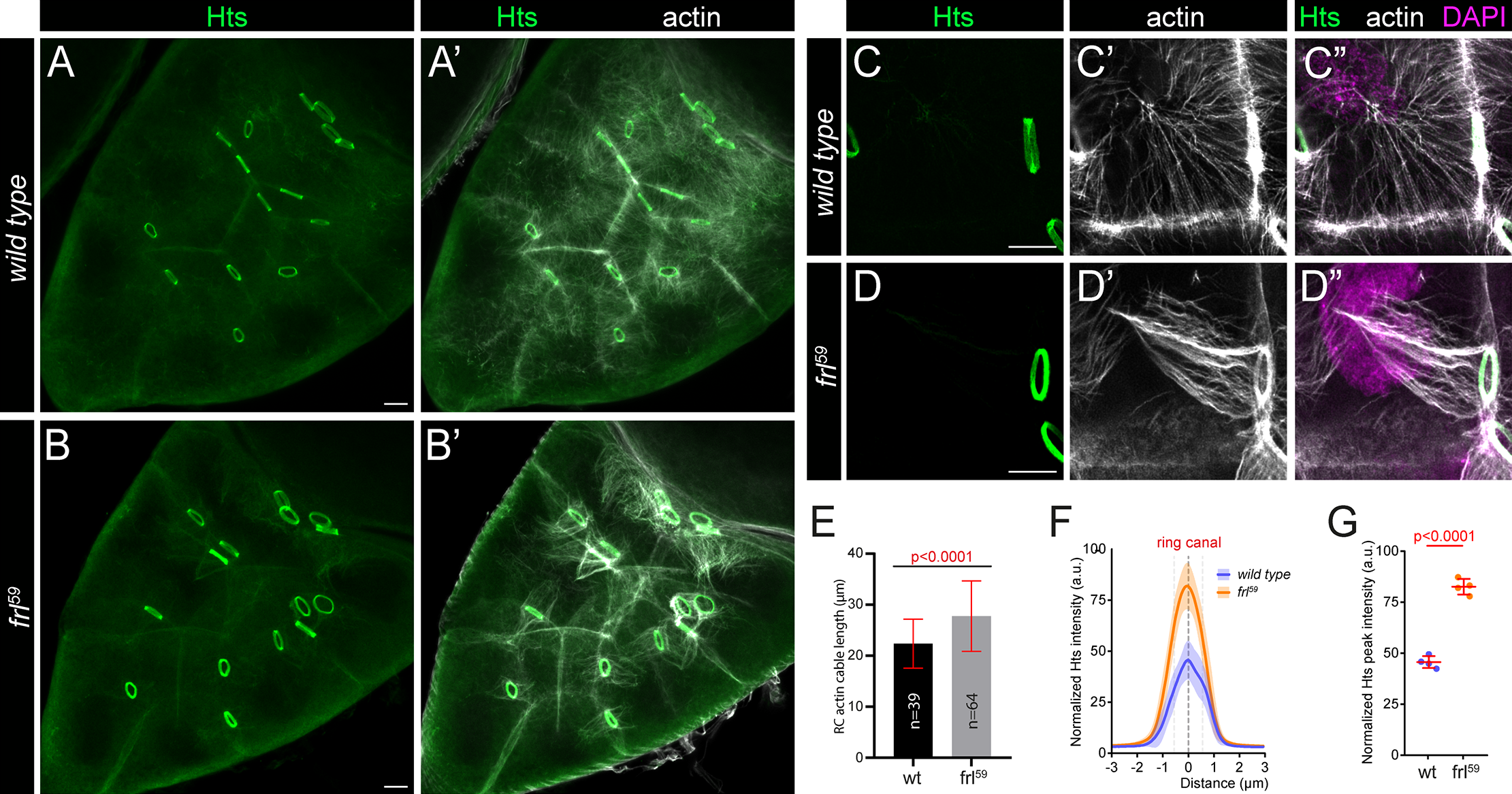

Supplement: S2 Fig — (TIF) [file pgen.1012042.s002.tif]

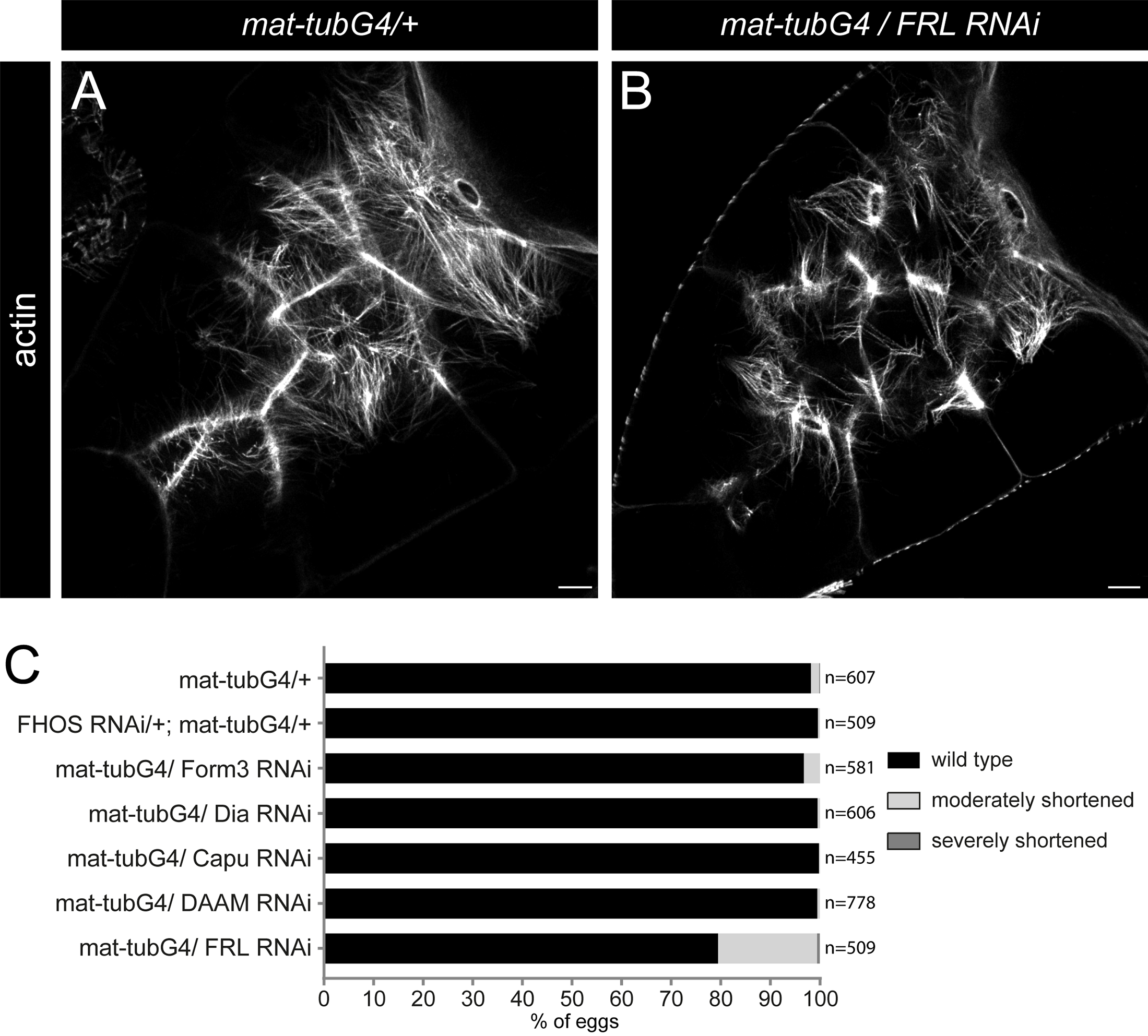

Supplement: S3 Fig — (TIF) [file pgen.1012042.s003.tif]

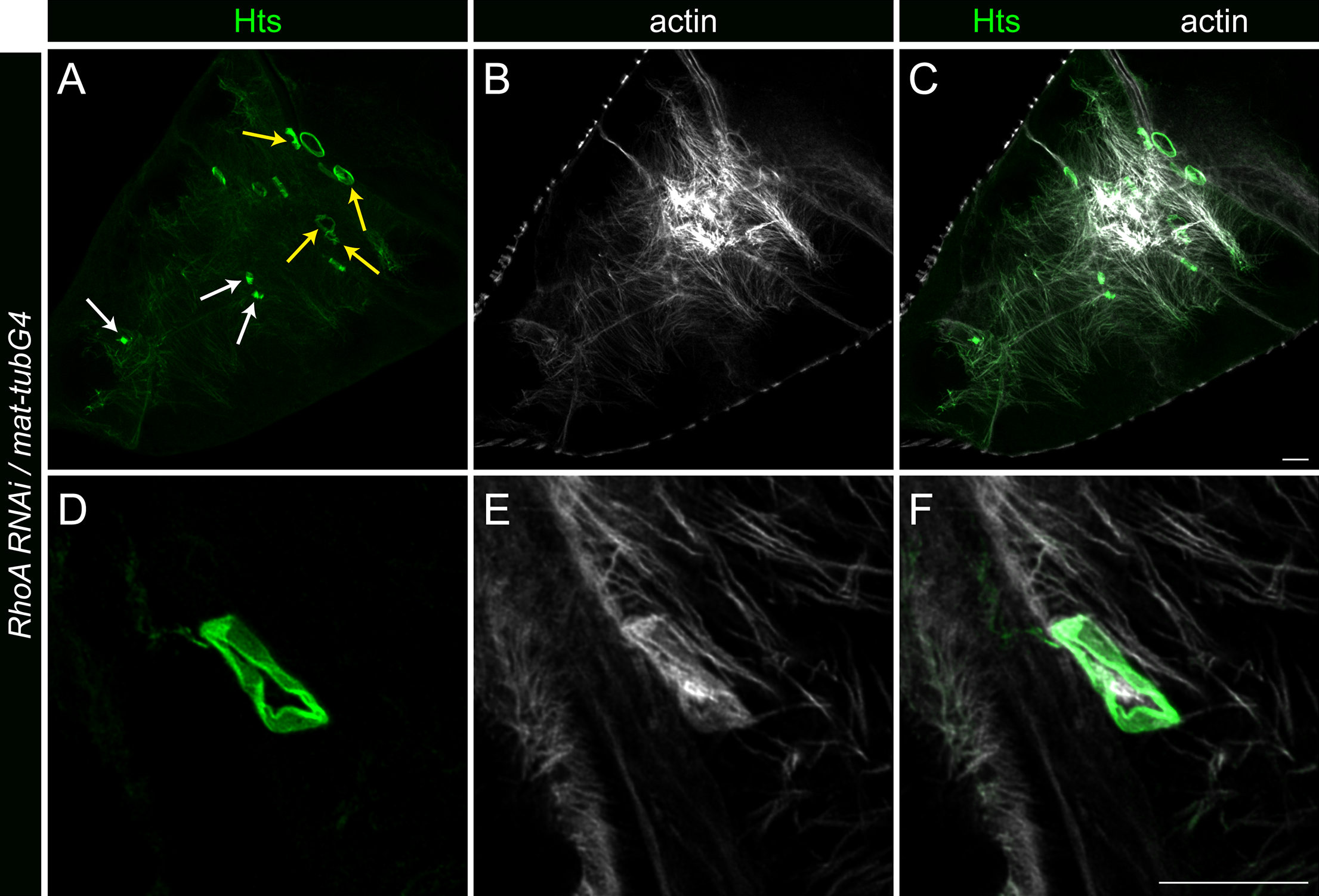

Supplement: S4 Fig — (TIF) [file pgen.1012042.s004.tif]

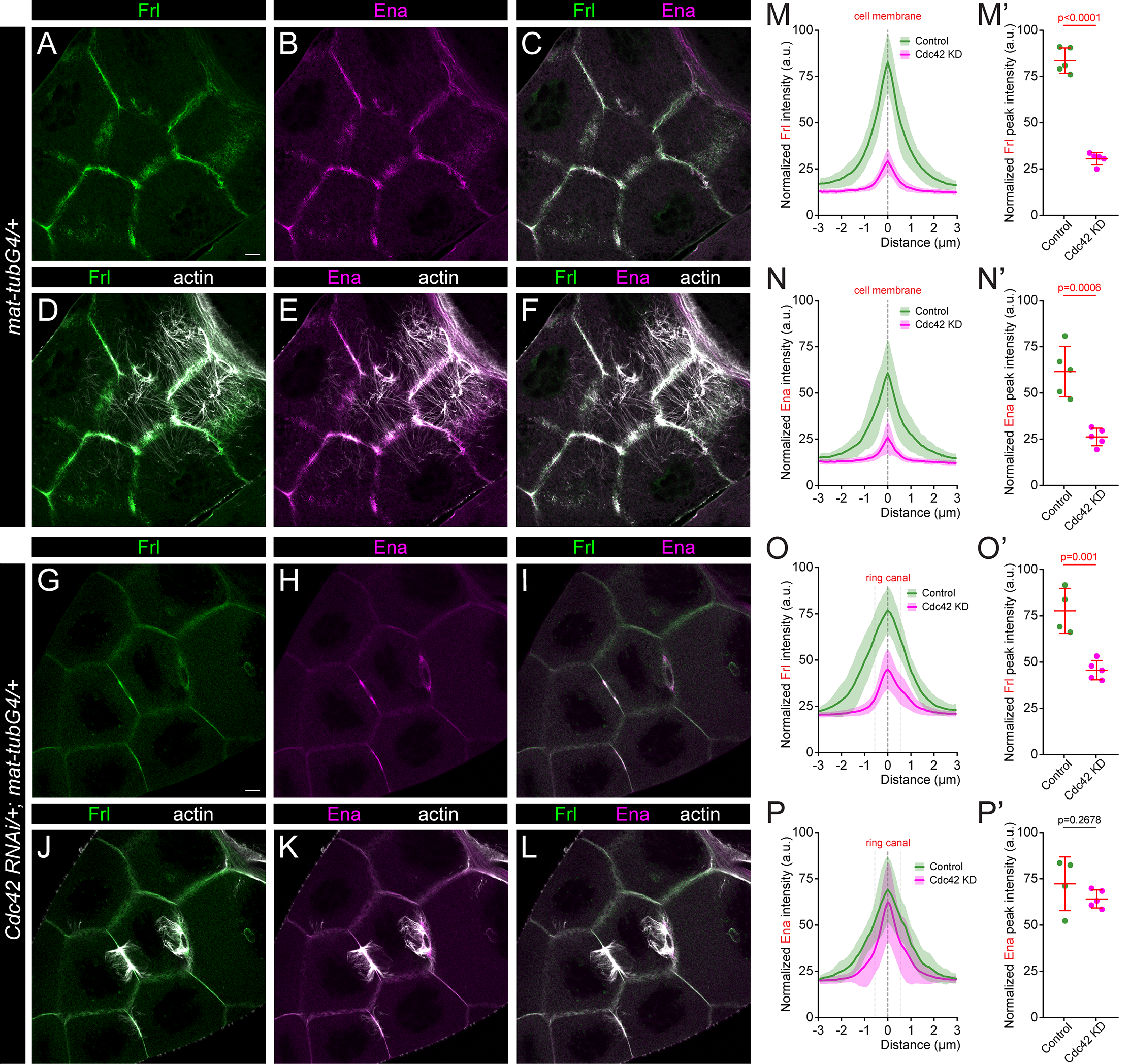

Supplement: S5 Fig — (TIF) [file pgen.1012042.s005.tif]

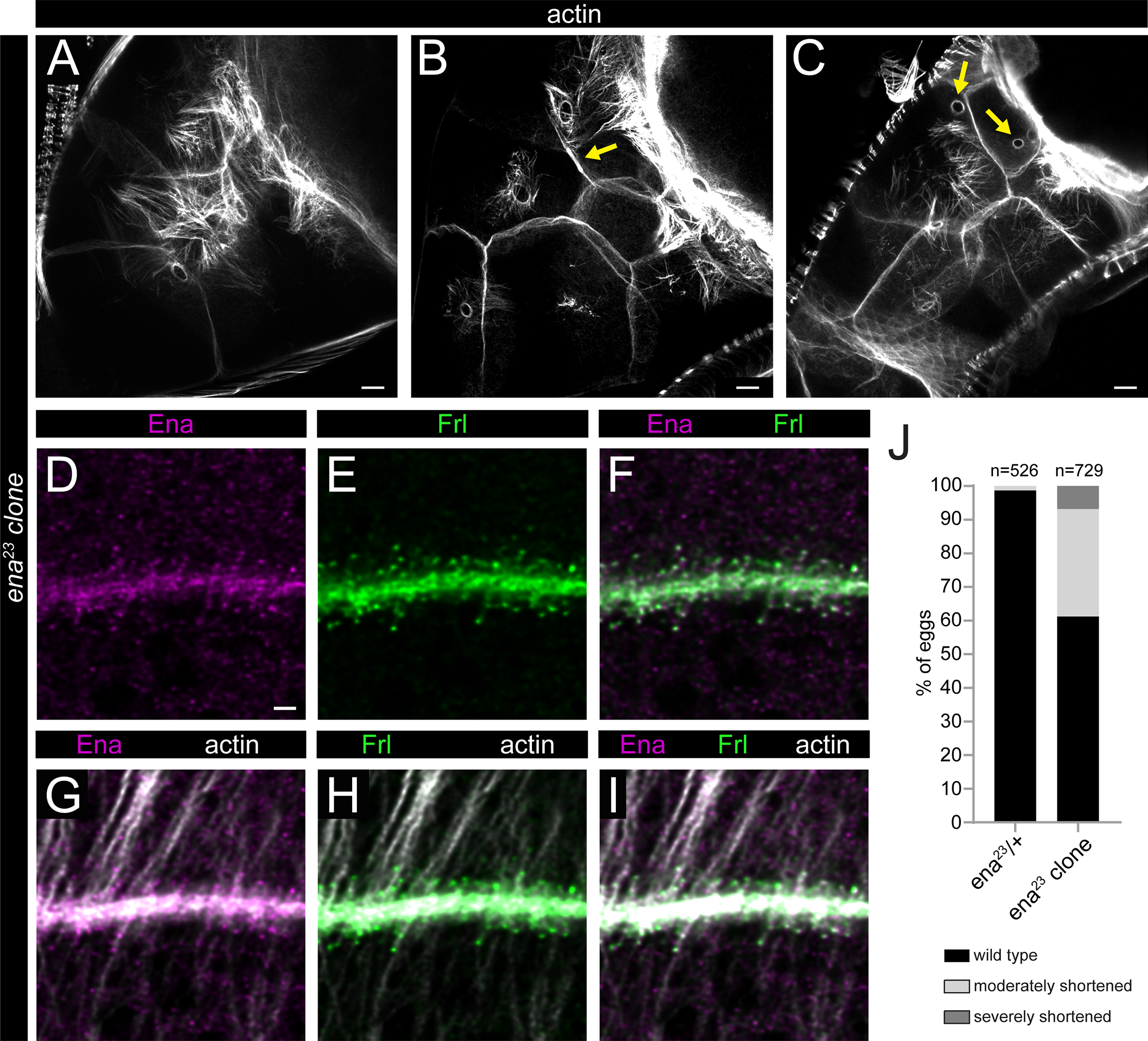

Supplement: S6 Fig — (TIF) [file pgen.1012042.s006.tif]

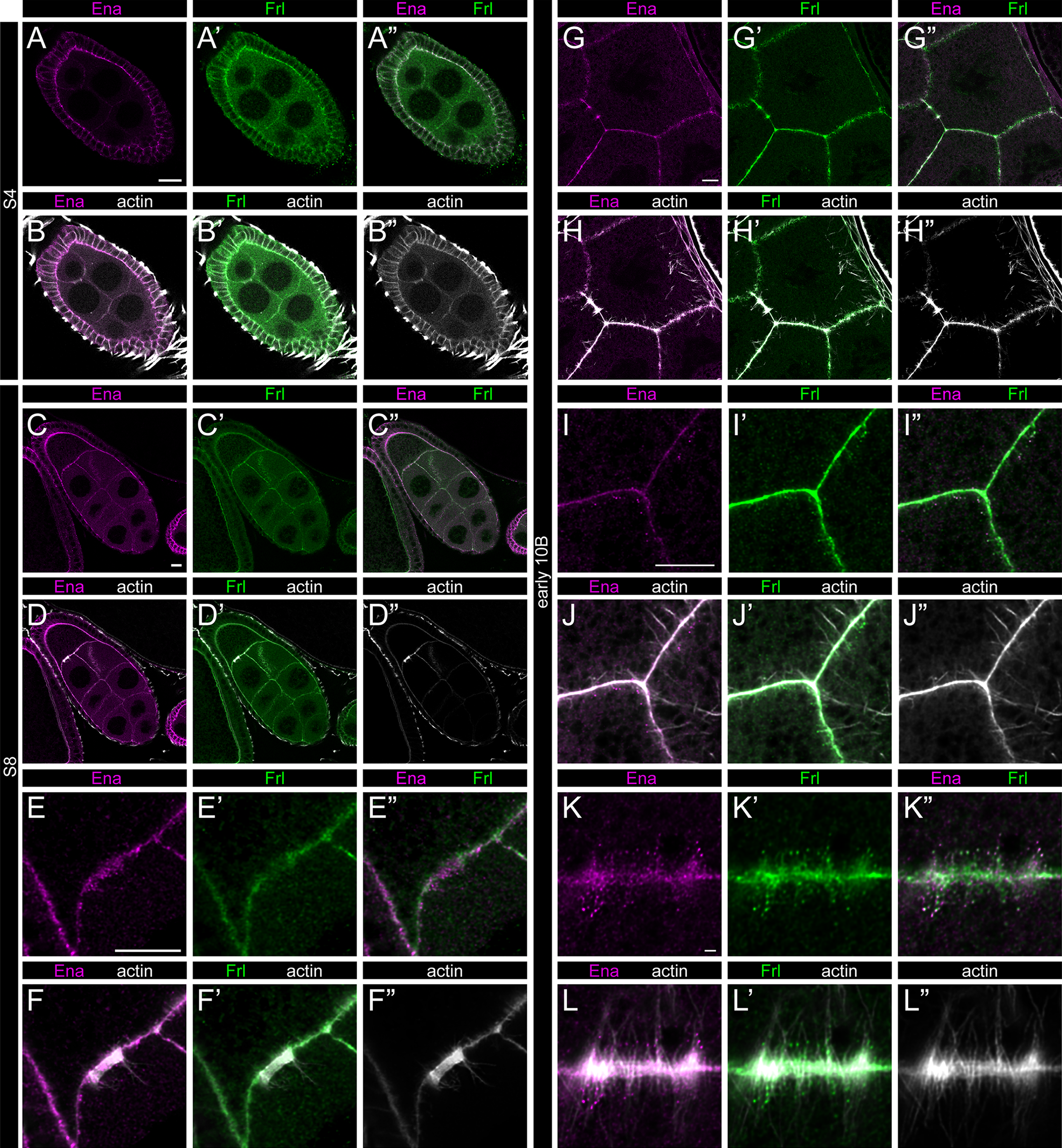

Supplement: S7 Fig — (TIF) [file pgen.1012042.s007.tif]

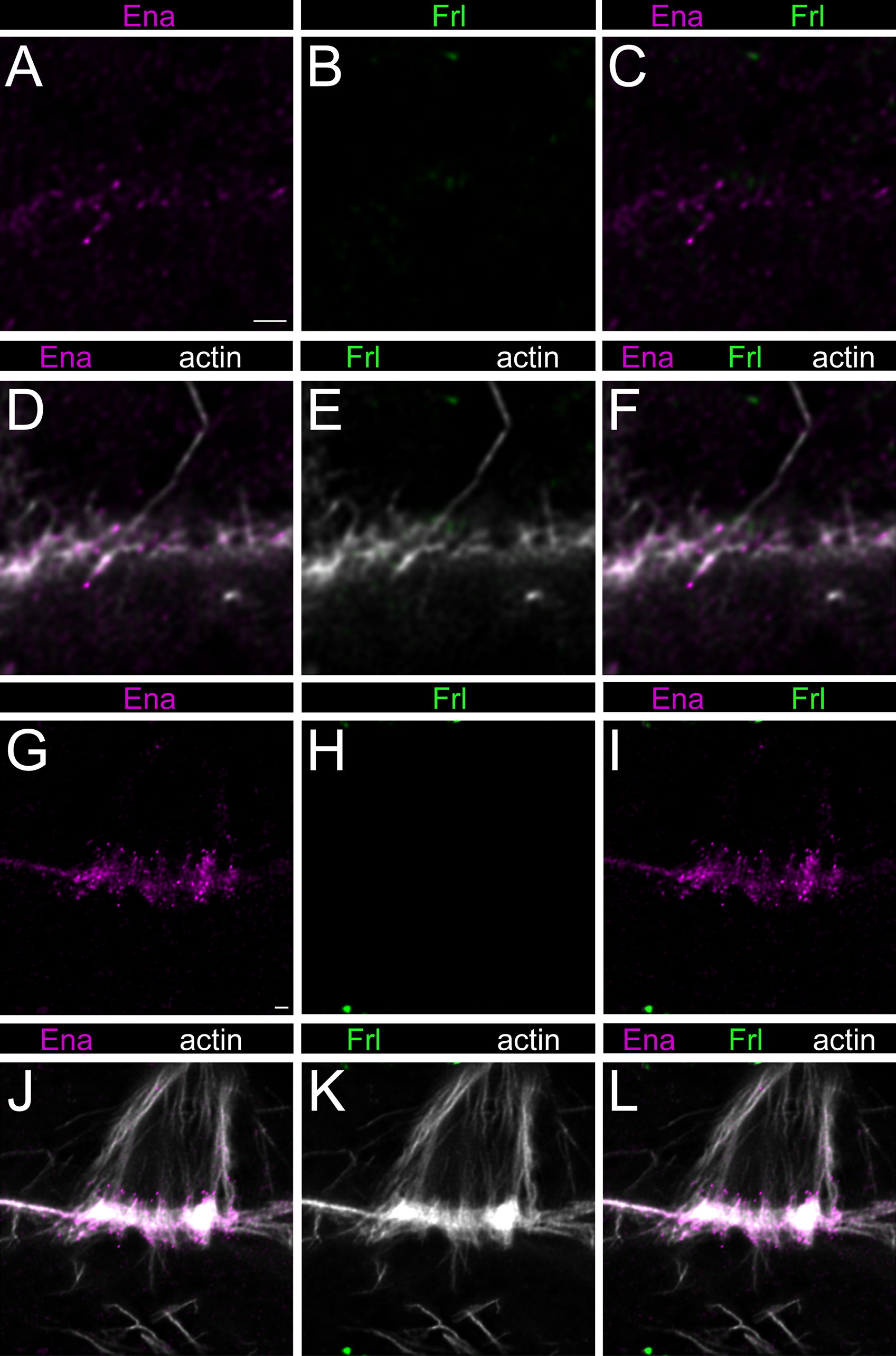

Supplement: S8 Fig — (TIF) [file pgen.1012042.s008.tif]

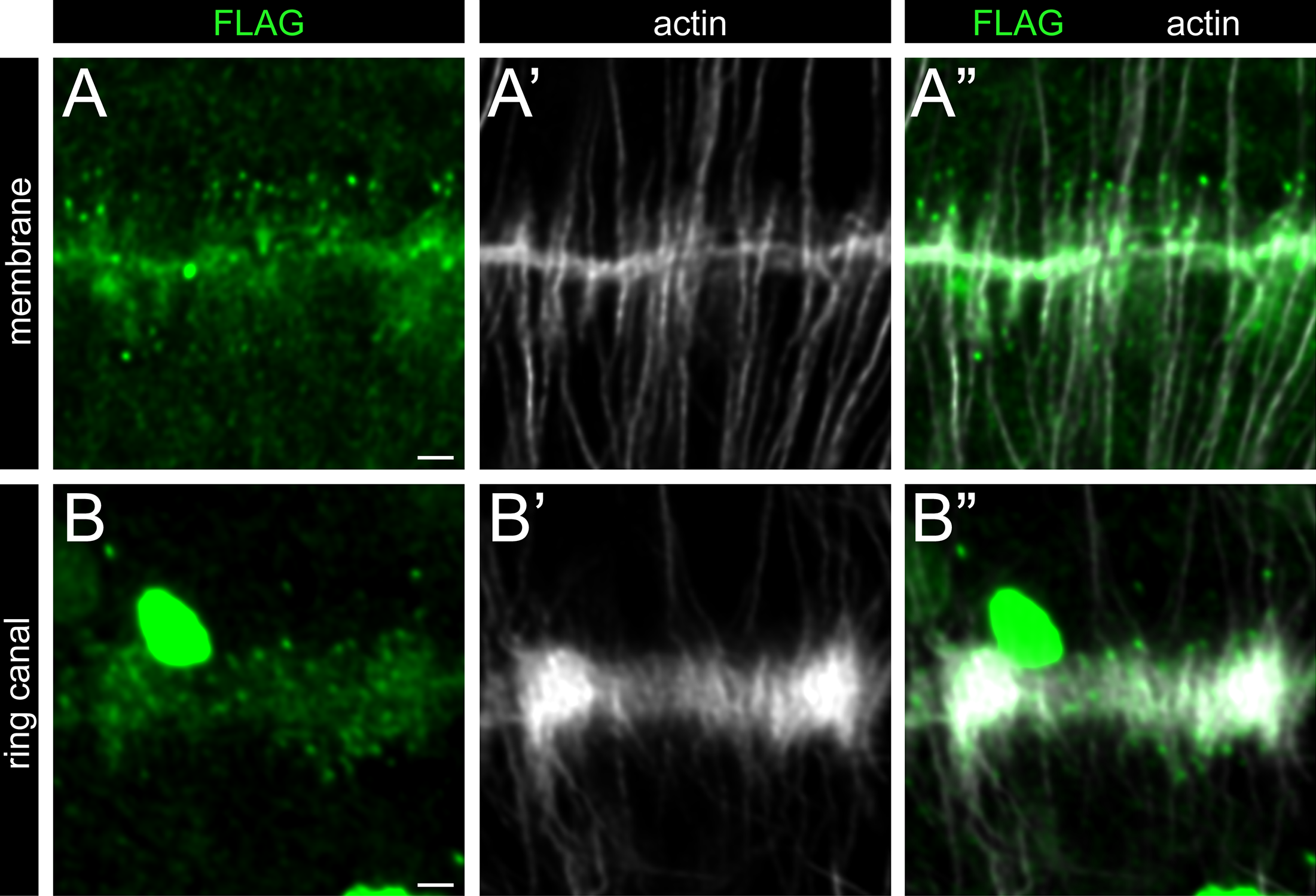

Supplement: S9 Fig — (TIF) [file pgen.1012042.s009.tif]

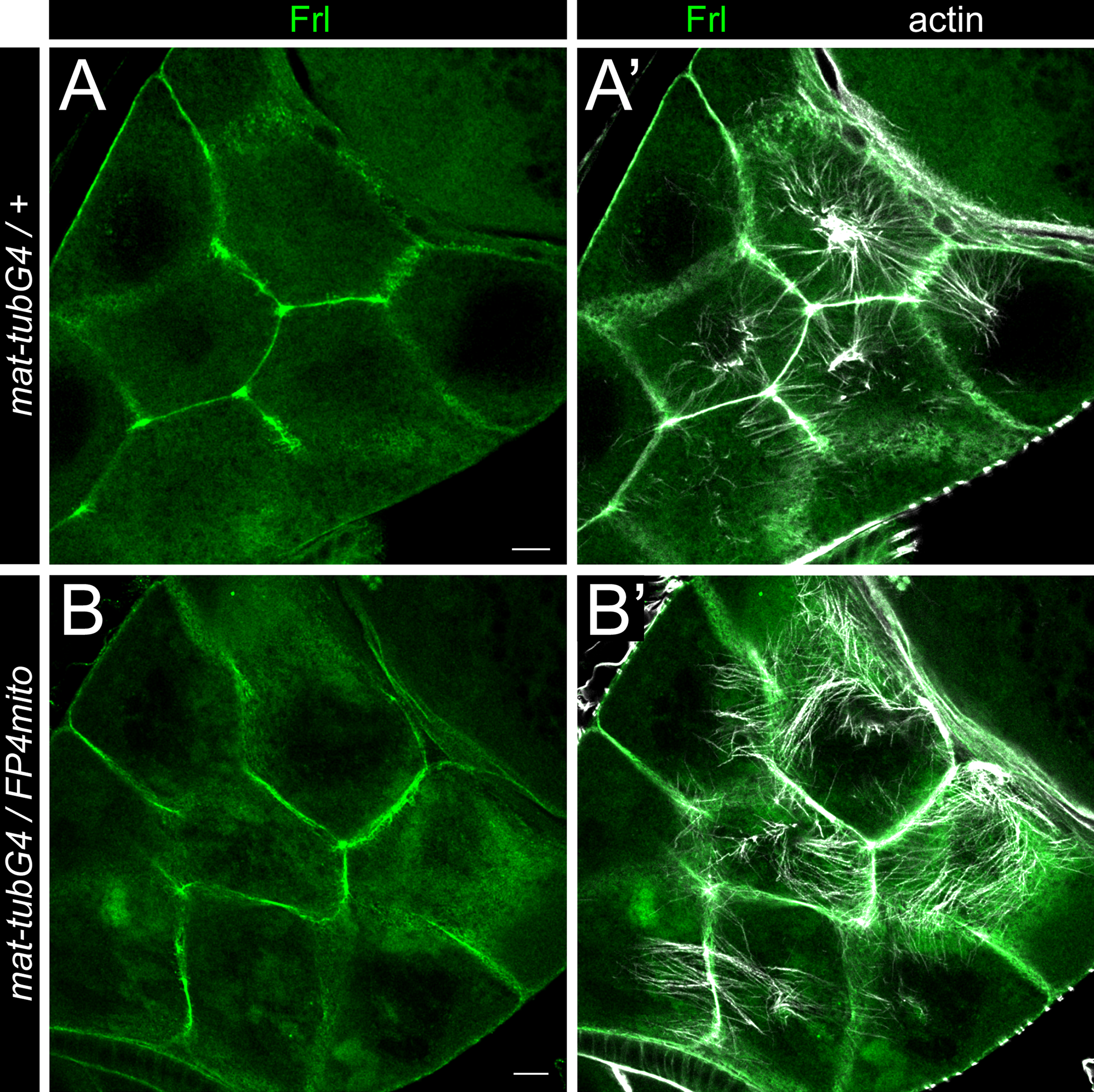

Supplement: S10 Fig — (TIF) [file pgen.1012042.s010.tif]
