# Supplementary material for: Spatially distinct FRL and Ena dependent actin networks coordinate nuclear positioning in Drosophila nurse cells
Source: PLoS Genet. 2026 Feb 9;22(2):e1012042. doi: 10.1371/journal.pgen.1012042 (PMC12915960; doi:10.1371/journal.pgen.1012042)
Supplement: Attachment — Submitted filename: Rebuttal2_MJGR.docx [file pgen.1012042.s012.docx]

December 17, 2025

Dear Editors,

Please find attached our revised manuscript (PGENETICS-D-25-00632) entitled " Spatially distinct FRL and Ena dependent actin networks coordinate nuclear positioning in Drosophila nurse cells".

We would like to thank the reviewers for their overall positive assessment of our paper. Reviewer #1 wrote that *”Overall, this is a nice characterization of the main regulators for the two populations of actin cables that hold and position the nuclei in the nurse cells. This manuscript contains an interesting collection of observations that will be of broad interest.*”, Reviewer #2 stated that “*This manuscript identifies for the first time FRL as the main actin regulator that forms cytoplasmic actin cables in Drosophila nurse cells and thus answers a longstanding open question.*” and that “*The provided data are based on sound genetic experiments.*”.

Additionally, the reviewers raised several constructive points of criticism as well, all of which are addressed point by point below. We believe that our experimental additions and corrections at numerous places have significantly improved our manuscript, and hope that you share this view.

With kind regards,

József Mihály

**Point by point response to the comments of Reviewer #1:**

Major points:

1. The manuscript often suffers from a lack of quantification of results. This quantification is particularly important for the characterisation of actin filaments, which is a central issue in this manuscript. The authors could draw inspiration from the quantification methods used in other studies on the same process, such as those in Logan et al. (doi:10.1242/dev.197442 ). For example, this would allow the rescue of the frl mutant with mat-tubG4-UAS-FRL to be characterised more accurately, as the actin distribution shown in Fig. 1E for the rescue is quite different to that shown in Fig. 1A for the control. More generally the absence of quantification for the actin cables applies to all figures.

Despite we agree that not for actin filament distribution, we need to point out that unlike stated, we did provide quantification of the results all over the manuscript. An entire set of quantification was included for egg laying/length, that we consider as the major biologically relevant readout of dumping efficiency, directly linked to nucleus positioning. Egg length can be measured with high precision in hundreds of individuals, resulting in statistically robust and meaningful data. As compared to that, because the egg chambers are quite thick (at least at stage 10B and further on) with a complex 3D organization, and actin distribution exhibits a high variability both among nurse cells of the same egg chamber and between egg chambers, imaging and reliable quantification of the nucleus positioning actin cables is inherently challenging. As to the inspiration one might get from the Logan et al., 2022 paper, it is important to see that most of the efforts in that work were focused on quantification of actin dynamics, and in fact the data on static or fixed actin filaments is very sparse in that paper, not to mention that the measurement method for these filaments, is rather poorly described. Nevertheless, we understand that this was a major concern of this Reviewer, and we now provide quantifications for the actin cables at numerous places, and our figures were modified accordingly.

Similarly, it would be valuable to quantify the positioning defects of the nucleus in NCs within the various genetic contexts described in the manuscript.

To further improve our paper as to quantifications, we provide measurements for positioning defects in Fig.6.

2. In the absence of an FRL, the actin cables connected to the ring canals are longer. However, quantifying the extent of these cables would improve the results (see Fig. 1). Furthermore, the ring canal in the frl59 mutant appears thicker and seems to present a brighter signal for Hts (Fig. S2). Quantification would help to address this point. It is possible that, in the absence of an FRL, the absence of Ena from the plasma membrane would trigger more actin at the ring canals and affect their morphology.

It was done accordingly, text and figures were modified correspondingly.

3. The authors analyse the distribution of FRL and Ena (see Fig. 2). Among the results, they show the presence of FRL-Ena puncta around the ring canals and propose that these dots are not directly connected to the ring canals, but rather to the area surrounding them. From the pictures presented in Figure 2, this is not obvious; colocalisation with a ring canal marker such as Hts and FRL would provide more conclusive evidence. In addition, it would be interesting to find out which part is connected to RCs and which part isn't.

The requested FRL-Hts double staining has been carried out. Unfortunately, due to a partial antibody crossreactivity (i.e. our anti-Rat secondary antibody reacts with Hts, while our anti-Mouse secondary does not recognize FRL; anti-Hts is a mouse, anti-FRL is a rat antibody), the quality of such a staining does not fully match our expectations, and we would avoid to include it in the main manuscript, but we include it here as additional information for the Reviewer. Besides that, we would like to add that an Hts, phalloidine double staining in *frl^59^* mutants (where the ring canal associated cables are present) also reveal that the RC cables are not linked to the central actin ring (shown in panels L-L” in the modified Fig. 1.).

*A wild type ring canal stained for actin (in grey), Hts (in green) and FRL (in magenta), stage 10B. A white arrow (in C, D, E) indicate a region with RC actin bundle tips, marked with FRL, which are clearly outside of the area of the RC proper, marked with Hts.*

4. Interestingly, the authors question the potential connection between GTPase regulation of actin assembly and FRL. They identified a genetic interaction between FRL and CDC42 and, based on this result, proposed that CDC42 is required for FRL activation. This remains a possibility that would be strongly supported by analyzing FRL distribution in nurse cells with cdc42 RNAi or mutant clones.

As suggested, FRL level was analyzed in *cdc42* mutant clones, as well as upon RNAi knockdown, and we detected a reduced FRL level in both conditions (Fig. S5). Whereas this observation is still not a direct evidence for Cdc42 being required for FRL activation, when combined with the genetic and phenotypic data, and the biochemical evidences, this piece of data provide further support for our proposal.

5. By combining efficient Ena delocalisation with the FP4mito and frl mutant, the authors reveal differential effects of the absence of FRL and Ena on actin cables and nuclear positioning. However, their conclusions suffer from a lack of accurate quantification.

It has been quantified as suggested, data is shown in Figs. 1, 5 and 6.

6. Importantly, the authors question the impact of FRL absence on Ena distribution. The results in Fig. 7 show that Ena distribution is affected at the plasma membrane. The labelling of Ena at the level of the ring canals also seems to be increased (Fig. 7B), which could be in line with the extension of the actin cable at the level of the ring canals observed in the absence of FRL. In this respect, it would be interesting to estimate the level of Ena at the ring canals in FRL mutants compared to controls.

It has been done. Results (shown in Fig. 7) and interpretations are now included at the relevant sites.

Minor points:

With the figures 1 and S1, the three different subpopulations of cytoplasmic actin cables derived from nurse cells-nurse cells borders, nurse cells-follicle cells borders and nurse cells-oocyte borders are not easily distinguishable. An inset with a zoom highlighting these three actin populations would help.

We thank for the useful comment, Fig. S1 was modified accordingly.

**Point by point response to the comments of Reviewer #2**:

The manuscript and the conclusions would improve by (i) more concise / precise wording and interpretation of achieved data; (ii) by further elaborating on the main conclusion that nuclear positioning in the nurse cells requires both types of actin cables (see below).

We made efforts to improve our wording, and carefully considered the specific comments of the Reviewer (see below)

1. Major issues:

1.1. The authors claim “nuclear positioning in the nurse cells requires the coordinated action of two spatially distinct actin networks” (e.g. lines 29-30).

1.1.1. I agree that the authors identified two actin structures, cytoplasmic cables and RC baskets/cables, that are somehow temporal and spatial distinct. But I am less convinced of both networks being required for nuclear positioning, more specifically of RC cables being required for nuclear positioning. An alternative explanation for the frl mutant phenotype could be an “out-of-balance” situation, in which the increased amount of unused Ena and/or other actin regulating proteins in frl[59] mutant nurse cells leads to an over-elongation of actin filaments forming actin baskets around RCs. These RC cables, by accident or not, are sufficient for some – I admit – remarkable nuclear positioning. So, the question arises: is there any evidence, that RC actin cables do localise nurse cell nuclei in a wild type background? Please elaborate and provide evidence or modify claim in manuscript.

We agree with the Reviewer that we did not provide a direct evidence for the role of the RC cables in the wild type situation. However, we would like to emphasize that it is not the example of the *frl^59^* mutant, rather it is the case of Ena depletion which directly underlines this conclusion. In this scenario most of the cytoplasmic cables are present but not the RC associated cables, and the nuclei often blocks the ring canals (Fig.5). This situation clearly indicates that presence of the cytoplasmic cables is not sufficient to hold the nuclei away from the RCs, to achieve that the RC associated cables are required too. Because in the wild type situation number of the cytoplasmic cables is only slightly bigger than upon Ena depletion, we have no reason to believe that it would make a significant functional difference. Thus, although in the case of the *frl* mutant we cannot dismiss the alternative explanation of the Reviewer, if we also consider the case of Ena depletion, we think it very unlikely that the cytoplasmic cables alone would be sufficient for normal positioning and the role we discovered for the RC cables would only be relevant in some mutant situations. Nevertheless, to handle this concern, we modified the text (in Results and Discussion) with the goal to improve clarity as to this issue. In addition, we now provide measurements for RC actin cable length in stage 10B wild type and *frl* mutant nurse cells (Fig. S2E), revealing that these actin bundles are long enough to span the distance between the ring canals and the nuclei, providing further support for our proposal.

1.1.2. line 135/136: please specify in the manuscript what makes RC associated cables a “distinct population” in stage 10B nurse cells. How are the authors able to distinguish RC actin cables from the other actin cables during stage 10B? By their location or by other features?

Indeed, we meant the difference in their location. We modified the text to improve clarity.

1.2. The discovery that the inactivation of Ena by FP4-mito leads to a phenotype opposite to frl[59] mutations supports the idea of two distinct subpopulations of actin cables. The use of FP4-mito might produce stronger phenotypes than the loss of Ena function, e.g. by mis-localising other proteins binding to FP4 motifs.

1.2.1. Could the authors provide further evidence (independent of FP4-mito), that lack of Ena function effects mostly RC actin cables, e.g. by analysing ‘classic’ ena mutations (e.g. ena[23])? And could the authors clarify to the reader the nature of the ena[23] mutation and include the information when discussing the results?

To adhere with this suggestion, we repeated the *ena^23^* germline clone analysis (already published in Gates et al., 2009), and we observed that this mutation mildly impairs the cytoplasmic actin cables, and we also detected an occasional loss or strong reduction of the RC associated cables. These findings are largely consistent with the former report of Gates et al., including the effect on the RC cables which is not discussed in their paper but the phenotype is visible on Figure 4, panel F3 where at least two RCs seemingly lack the associated actin cables. These RC cable phenotypes are weaker than upon the FP4-mito mediated depletion of Ena. However, unlike in the case of Ena depletion where Ena level appears to be severely reduced (causing a nearly protein null situation in the cytoplasm), *ena^23^* is clearly not a null allele because the Ena protein can still be detected in the germline clones with a distribution pattern that is similar to wild type (Fig. S6). Thus, the hypomorphic nature of *ena^23^* and the largely normal protein accumulation pattern provide a conceivable explanation for the phenotypic difference as compared to Ena depletion, and overall the data support further for an Ena role in RC actin cable formation.

As requested, we included information on *ena^23^* which carries two mutations, N379F and K636STOP, resulting in a truncated protein largely lacking the tetramerization domain but retaining the actin binding region.

1.2.2. The use of FP4-mito leads to the loss of RC baskets in early egg chambers (Fig. S1 G-I). It appears to me that also filamentous actin structures at the oocyte cortex and at the membrane between oocyte and nurse cells are reduced / gone. Could it be that FP4-mito reduces generally filamentous actin and that later, i.e. stage 10B, FRL-dependent cables arise independently of Ena (or independently of whatever is recruited by FP4-mito)? Please comment and consider in Discussion.

Indeed, we cannot exclude the possibility raised by the Reviewer that Ena has an early role (at the RCs, and presumably at other nurse cell plasma membrane locations), whereas the FRL dependent cables not only arise later but also independent of Ena. Although the colocalization of FRL and Ena at the tip of the membrane pits where the nucleus positioning cytoplasmic cables form in stage 10B might indicate that these two proteins work together in the wild type situation, we now consider both possibilities in the revised Discussion.

1.3. line 177 “the lack of redundancy”: could the authors provide evidence and comment in the manuscript on efficiency of the RNAi lines and on timing? Could it be that the KD using RNAi is not early enough to affect the formation of the RC actin basket? The different strengths of Ena-RNAi and of FP4-mito effects could indicated that the RNAi needs more time for a stronger cellular effect.

Whereas egg chamber specific quantitative measures are not available for efficiency of the RNAi lines, we now provide more information on the RNAi lines (in the Materials and methods section) demonstrating their efficacy in other tissues. As to timing, we used an early driver for all knockdown experiments, and because the KD of Cdc42 worked very efficiently with the same driver (Fig. 4), we consider it as a strong indication against the timing factor, even if formin RNAi efficiencies remain less clear.

1.4. line 192, chapter “Cdc42 and FRL work together in the nurse cells”

1.4.1. The conclusion “activity of FRL is regulated by Cdc42” (line 98/99 or similarly line 192) is – I guess – based on the genetic interaction of frl[59] mutations that enhance dominantly a weaker allele of Cdc42 in respect to egg lengths. Even though the literature supports this notion, the authors could substantiate their conclusion by e.g. testing how Cdc42 affects e.g. FRL (and Ena) localisation – as potential read out(s) of FRL activity. Please include the data in the manuscript. Alternatively, the conclusion should be adequately phrased. Please adapt similarly line 214 “Cdc42 is required for FRL” and line 219.

We thank the Reviewer for this suggestion. We tested how Cdc42 affects FRL and Ena levels, and we detected a reduction for both proteins at the plasma membrane (data are included in Fig S5), which further substantiates our conclusion.

1.4.2. Fig. 4: Please add the phenotypes of Cdc42 [2] enhancement by the frl[59] mutation to the images and Cdc42[4] to the egg lengths quantification.

The requested image is now included in Figure 4. Unfortunately, egg length quantification for cdc42[4] clones is not a feasible experiment because eggs are hardly laid.

1.4.3. Fig.4 D: please comment in the manuscript on the stronger phenotype of Cdc42 knock down compared to frl[59] mutations. Does KD of Cdc42 affect not only FRL but also Ena? Provide evidence.

While we have commented on this finding already in the first version of our manuscript (lines 209-216), inspired by the Reviewer, we added more details on this issue. Also, because the KD of Cdc42 affects both FRL and Ena levels, this question is now discussed more explicitly in the revised text.

1.5. lines 264/265 “selectively affects the accumulation and localization of Ena in the developing egg chambers”.

1.5.1. Fig.7 A, B: please indicate areas with reduced Ena and provide quantification; include images showing the localisation of Ena at plus ends of RC cables and cytoplasmic actin cables lacking FRL (with quantification).

The figure was modified accordingly, including the requested quantifications (Fig.7C-D’ and S8).

1.5.2. In Results and Discussion, please bring together Ena and FRL localisation with their described functions: are they differentially localised / expressed in nurse cells during the time course of oogenesis? Provide supporting evidence.

As suggested, the localization of FRL and Ena has been examined in more details to cover the entire course of oogenesis. The results of this analysis (shown in Fig. S7) are described in the revised manuscript, and also mentioned in the Discussion.

1.5.3. line 270 “overexpression of Ena in frl59 mutants had no such an effect (Fig. 7E)”. Could the authors show that overexpressed Ena did indeed localise to plasma membranes? Otherwise, a rescue is not expected.

We carried out the requested control experiment, leading to the conclusion that overexpression of the Ena::FLAG protein results in a strong membrane accumulation (much alike localization of the endogenous wild type protein). This new data is now shown in Fig. S9.

1.6. When revising the Discussion:

1.6.1. Please provide a model of the functions of FRL (nucleator & elongator) and Ena (elongator) in the different subpopulations of actin cables, including how they depend on each other or not, early vs late cables, and speculate on the functionality of remaining actin cables lacking Ena or FRL.

Because we felt that our experiments did not specifically address this question, we initially avoided a lengthy discussion on this matter. In the revised Discussion we considered the possible molecular mechanisms and differential contribution from FRL and Ena in a lot more details than in the original version of the manuscript. However, in the absence of a true molecular dissection of these functions, any model would remain so speculative or so general that we do not feel them particularly beneficial or necessary to help understanding.

1.6.2. A more extensive discussion of evolutionarily conserved / non-conserved mechanisms of FRL / Ena functions for the formation of filopodia-like structures would make the relevance of the results more apparent to a general audience.

We thank the Reviewer for this suggestion as well. A new paragraph has now been added to discuss the potential relevance of our new findings in light of the known mechanisms of the formation of filopodia-like structures.

2. Minor issues

2.1. Please define the types of actin cables in the beginning and use defined terms consistently in the entire text, e.g. - just one possible example - RC cables vs. cytoplasmic cables (which are oocyte cables, nurse cell cables, and follicle cables; ref. 20 of manuscript). Define what means “membrane associated”; are not all actin cables associate with a membrane? In my opinion the term “RC actin” refers to the actin of RCs proper but not to actin cables associated with RCs (e.g. line 215, line 373; similar: line 291/292, line 394).

A fair enough comment, that prompted us to correct the manuscript at several places to use the terms consistently throughout the text.

2.2. The graphs showing the quantifications of egg lengths (Fig.1 F, Fig.3 G, Fig.4 D &E, Fig.5 O Fig.7 E, Fig.8 E) are not intuitive to me as they show the percentage of eggs within a specific class of lengths; they do not show explicitly the lengths of eggs. Thus, claims like “produce shorter eggs” (e.g. line 231) are not ‘directly’ visible. Showing absolute lengths would be less simplified and more intuitive.

We disagree with this comment as showing the average of the absolute length would often mask the real differences between the different genotypes. We believe that in our case the (simplified) length distribution charts are clearly more informative than averaging the absolute lengths. We provided the quantification for all genotypes, and the length categories were unambiguously defined in the second sentence of the Results. Now, we included a set of embryo pictures in Fig. 1G to illustrate the length differences with raw data.

2.3. In all above-mentioned graphs, the axes need to be labelled properly as % of eggs.

Done.

2.4. For all figures: please indicate the exact stage of each egg chamber shown.

Done.

2.5. line 229 “Ena is absent from the nurse cell plasma membranes and the ring canals”. Please specify imaging conditions (include in methods): were these identical to e.g. Fig1 / Fig.2, despite Ena being strongly recruited to mitochondria?

As assumed, staining conditions were indeed identical, and it was already described in the original version of the manuscript.

2.6. line 238/239 and Fig.6 A: specify which actin was quantified how (include in methods).

Done (included in Methods).

2.7. Please indicate in methods the isoform of Ena that was used for the UAS-Ena-FLAG construct.

Done.

2.8. line 310 “retains the nucleus in (roughly) the middle of the nurse cell during dumping”. This is not correct: actin cables push nuclei to the side of nurse cells (side of squamous follicle cells). Please correct.

Corrected accordingly.

2.9. Define terms like “mildly” (lines 235), “largely” (lines 236), and “more frequently“ (line 240, numbers for each egg chamber?) and provide quantification.

Quantification is now provided, and the loosely defined terms are underscored by data.

2.10. line 295 “ena; frl double mutant combinations”. Please change to an accurate genetic description.

Done.

2.11. line 348/349: please specify used deconvolution tools (include in methods).

It is included in the originally submitted manuscript (lines 466-467).

2.12. phrase lines 351 – 355: the first process is not a process during dumping; please correct sentence.

The sentence was relocated and rephrased, hope it reads correct now.

2.13. wording: nurse cells “feeding” the oocyte (line 67) => “provide”

Corrected accordingly.

2.14. wording: the “intense” transport of materials towards the oocyte (line 307, 356) => “dumping” or “fast, un-selective” / “bulk” transport

Corrected accordingly.
